# Serum Soluble HLA-E in Melanoma: A New Potential Immune-Related Marker in Cancer

**DOI:** 10.1371/journal.pone.0021118

**Published:** 2011-06-21

**Authors:** Mathilde Allard, Romain Oger, Virginie Vignard, Jean-Michel Percier, Giulia Fregni, Aurélie Périer, Anne Caignard, Béatrice Charreau, Karine Bernardeau, Amir Khammari, Brigitte Dréno, Nadine Gervois

**Affiliations:** 1 UMR INSERM, U892, Nantes, France; 2 University Nantes, Faculté des Sciences, Nantes, France; 3 Institut Cochin, University Paris Descartes, INSERM U1016, CNRS UMR 8104, Paris, France; 4 UMR INSERM, U643, Nantes, France; 5 Recombinant protein production facility of the IFR 26, Nantes, France; 6 Unit of Skin Cancer, Centre Hospitalier Universitaire de Nantes, Nantes, France; 7 GMP Unit of Cellular Therapy, Centre Hospitalier Universitaire de Nantes, Nantes, France; Centre de Recherche Public de la Santé (CRP-Santé), Luxembourg

## Abstract

**Background:**

Tumor-derived soluble factors, including soluble HLA molecules, can contribute to cancer immune escape and therefore impact on clinical course of malignant diseases. We previously reported that melanoma cells produce, *in vitro*, soluble forms of the non-classical MHC class I molecule HLA-E (sHLA-E). In order to investigate sHLA-E production by various tumors and to address its potential value as a tumor-associated marker, we developed a specific ELISA for the quantification of sHLA-E in biological fluids.

**Methodology/Principal Findings:**

We developed a sHLA-E specific and sensitive ELISA and we showed that serum sHLA-E levels were significantly elevated (P<0.01) in melanoma patients (n = 127), compared with healthy donors (n = 94). sHLA-E was also detected in the culture supernatants of a wide variety of tumor cell lines (n = 98) including melanomas, kidney, colorectal and breast cancers. Cytokines regulation of sHLA-E production by tumor cells was also carried out. IFN-γ, IFN-α and TNF-α were found to upregulate sHLA-E production by tumor cells.

**Conclusions/Significance:**

In view of the broad tumor tissue release of HLA-E and its up-regulation by inflammatory cytokines, sHLA-E should be studied for its involvement in immune responses against tumors. Interestingly, our results demonstrated a positive association between the presence of serum sHLA-E and melanoma. Therefore, the determination of sHLA-E levels, using ELISA approach, may be investigated as a clinical marker in cancer patients.

## Introduction

Evidences accumulated demonstrate the ability of the immune system to identify and destroy malignant cells, in order to prevent tumor development. This process called cancer immunosurveillance, is based on the joined action of the effectors of innate (NK, NKT cells, γδ T cells, and dendritic cells) and adaptive (antigen-specific T and B cells) immunity to sense and eradicate nascent-transformed cells. However, despite the existence of well-defined immunogenic tumor antigens, and even in the presence of tumor-antigen-specific cytotoxic T cells, the immune system does not seem to be fully effective in eradicating tumors [1].

Several mechanisms have been implicated in tumor immune escape, including structural and functional alterations of the Human Leukocyte Antigens (HLA), which represent frequent events in cancers. This include classical HLA class I antigen (HLA-A, -B, -C) loss or downregulation and aberrant expression of non-classical HLA class I antigens (HLA-E, -G), providing mechanisms leading to a decrease in recognition and destruction of tumor cells by immune cytotoxic effectors (mainly CTL and NK cells) [2]. Interest in non-classical HLA molecules has been stimulated by the demonstration that they may contribute to NK and T cell tumor cell escape through their interaction with NK inhibitory receptors [3]–[6]. Furthermore, production of non-cell bound soluble HLA (sHLA) molecules has also been described and may represent an alternative strategy for cancer immune escape [7], [8]. In support of this hypothesis, sHLA molecules have been shown to induce *in vitro* inhibition and/or apoptosis of CTL and NK [9], [10]. Moreover, increased serum levels of classical and non-classical sHLA have been described in several malignant diseases, and their prognostic relevance is suggested by statistically significant association with high stage disease or with particular clinical course in various malignancies [11].

We previously observed, in collaboration with pathologists, a frequent expression of HLA-E by melanomas and colon carcinomas [12]. Studies from our group and others supported an immunosuppressive potential of this expression, through engagement of the CD94/NKG2A inhibitory NKR on cytotoxic cells (NK and CD8 TIL) by tumor cell membrane HLA-E [13]–[16]. We also reported that melanoma cell lines can produce soluble forms of HLA-E (sHLA-E) *in vitro*, by protease dependant shedding of surface molecules, and that this production was increased by IFN-γ [12].

The aim of the present project was to develop an ELISA assay for the detection and the quantification of sHLA-E in biological fluids, to validate its specificity and, if adequate, to address the clinical significance of sHLA-E levels in the blood of melanoma patients.

## Results

### Development of an HLA-E specific ELISA

**Figure 1:**
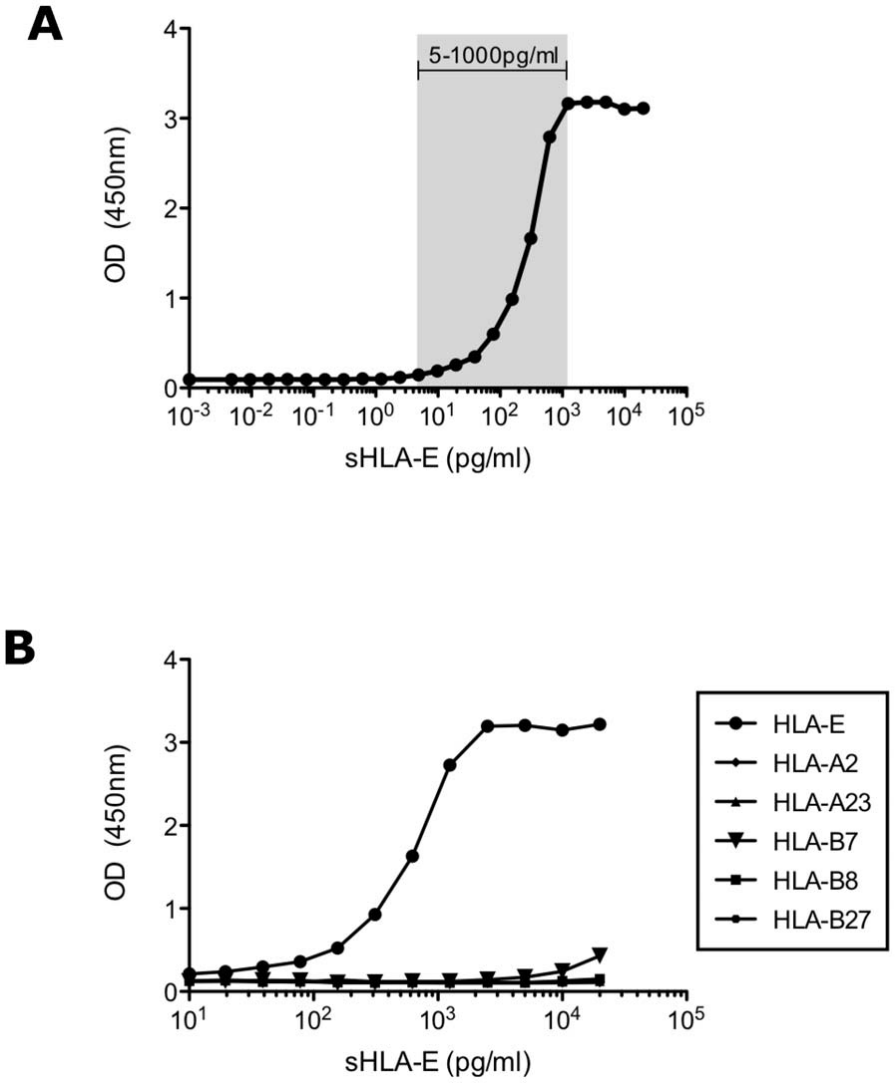
Sensitivity and specificity of sHLA-E ELISA. A/ Detection of sHLA-E using serial dilutions of recombinant HLA-E monomer. The grey area indicates the range of measurable sHLA-E levels. B/ Determination of the HLA-E binding specificity. Serial dilutions of HLA-A2, -A23, -B7, -B8 and -B27 recombinant soluble monomers have been tested in comparison with recombinant HLA-E monomer.

To detect and quantify sHLA-E in biological samples, we develop a sandwich ELISA using noncompeting solid capture and biotinylated detection anti-HLA-E monoclonal antibodies MEM-E/08 and MEM-E/07 respectively. First a concentration range of a purified recombinant soluble HLA-E (rsHLA-E) was tested. A shown on Figure 1A, rsHLA-E was detected at a minimum concentration of 5 pg/ml. Given the reported cross-reactivity of both anti-HLA-E Abs with four HLA class Ia allelic forms: HLA-A23, -B7, -B8 and -B27, we then checked their detection using our designed ELISA assay. Among recombinant soluble HLA-A23, -B7, -B8 and -B27 as well as rsHLA-A2 used as negative control, only a weak signal was obtained with rsHLA-B7 at the maximal dose 20 ng/ml (Fig. 1B).

These results led to the conclusion that the sandwich ELISA described here is highly specific and sensitive for HLA-E and could be used as a screening method for the detection of sHLA-E in biological samples.

### Increased soluble HLA-E in sera of melanoma patients

**Figure 2:**
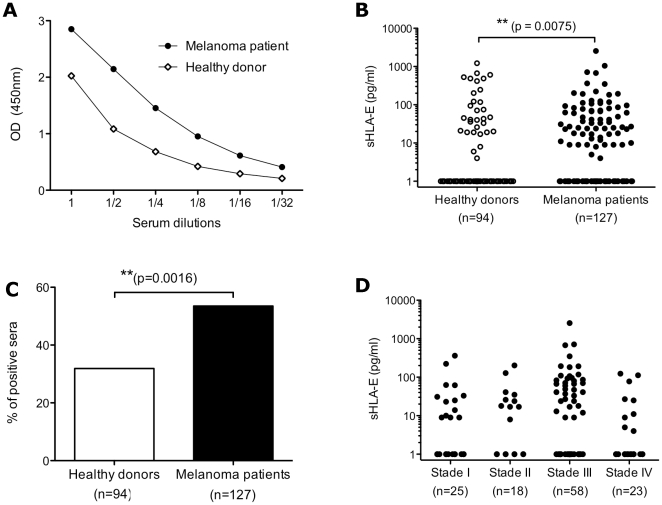
Analysis of sHLA-E in sera of healthy controls and melanoma patients. A/ Illustrative detection of sHLA-E using serial dilutions of two serum samples by ELISA. B/ Distribution of soluble HLA-E concentrations in sera of healthy controls and melanoma patients. P-value indicates the difference between the two groups. C/ Percentages of positive sHLA-E sera (sHLA-E≥5 pg/ml) in healthy donors and melanoma patients. D/ Distribution of soluble HLA-E concentrations in sera of melanoma patients with regard of tumor stages.

We screened sera from 94 healthy donors and 127 melanoma patients without current therapy for the presence of sHLA-E (Table 1). We detected sHLA-E in serum samples from both healthy and patient donors in a dilution-dependent manner (Fig. 2A) demonstrating that this ELISA could be used to quantify sHLA-E in sera samples. Considering the possible incidence of age and the gender, no significant differences in sHLA-E levels were observed (data not shown). The individual values of serum sHLA-E are presented on a dot-plot using a log scale (Fig. 2B) and means, medians and ranges are indicated on Table 2.

**Table 1:** Characteristics of healthy donors and melanoma patients studied.

	N° of cases	Age			Gender	
		Mean	Median	Range	Male	Female
Healthy donors	94	42.5	44	19–67	46	48
Melanoma patients	127	55	54.5	20–88	81	46

**Table 2:** sHLA-E in sera of healthy donors and melanoma patients studied.

	N° of cases	sHLA-E (pg/ml)			Positive sHLA-E sera (%)*
		Mean	Median	Range	
Healthy donors	94	61.6	0	0–1224	31.9
Melanoma patients	127	72.6**	9	0–2544	53.5**
Stage I	25	34.9	9	0–361	52
Stage II	18	28.9	12.5	0–202	55.6
Stage III	58	109.7**	21	0–2544	60***
Stage IV	23	17.2	0	0–124	34.8

**p<0.01,

***p<0.001.

p-values indicate the differences between healthy controls and the patient groups.

*Sera are considered as positive if sHLA-E levels are higher than or equal to 5 pg/ml.

In melanoma patients, serum levels of sHLA-E (median [range]) were significantly increased compared with those in healthy controls (9 pg/ml [0–2544] vs 0 pg/ml [0–1224], respectively, p<0.001). To quantify the frequency of sHLA-E-positive sera, we take a cut-off value of 5 pg/ml. This analysis revealed that in the group of 94 healthy donors, sHLA-E could be detected in 30 sera (31.9% of total). In contrast, in melanoma patients, 68 out of 127 sera (53.5% of total) contained sHLA-E (Fig. 2C). Thus, percentages of positive sera were significantly higher in melanoma patients compared to healthy donors (p<0.001). Subgroup analysis considering AJCC stages (American Joint Committee on Cancer, http://www.cancerstaging.org/) was then performed. No relationship of serum sHLA-E levels was found with respect to tumor grading (Fig. 2D). However, as shown in Table 1, sHLA-E levels were significantly increased in stage III melanoma patients compared with healthy donors (p<0.0001).

Together, these results validated an ELISA for the determination of sHLA-E in human serum and demonstrated that sHLA-E is significantly increased in sera of melanoma patients.

### Production of soluble HLA-E by a diverse panel of human tumor cell lines

**Figure 3:**
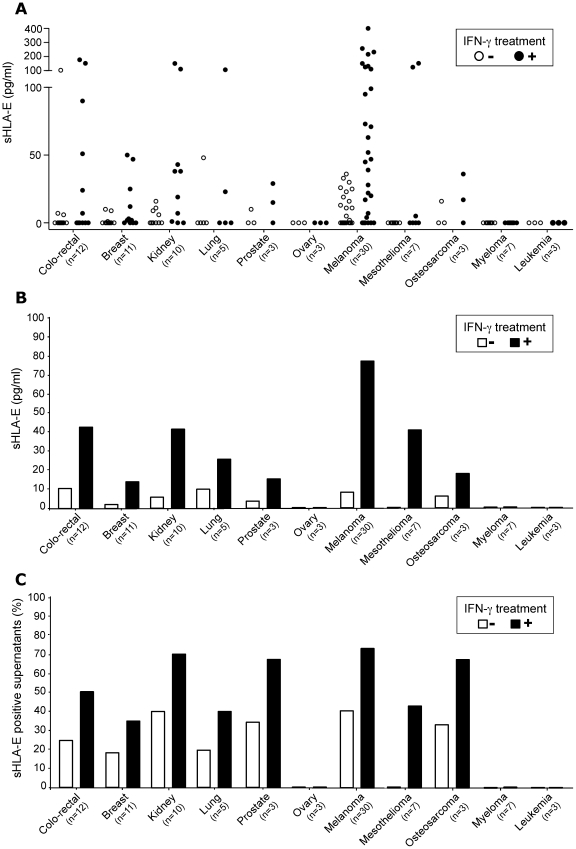
Analysis of sHLA-E production by tumor cell lines. Analysis of soluble HLA-E concentrations in supernatants of tumor cell lines treated or not by IFN-γ with regard of tumor origins: distribution of individual concentrations (A), mean levels (B) and percentages of positive supernatants (sHLA-E≥5 pg/ml) (C).

We analyzed the production of soluble HLA-E by 98 different established human tumor cell lines, representing solid tumors as melanomas (n = 30), carcinomas (cancers of the lung (n = 5), colo-rectum (n = 12), kidney (n = 10), ovary (n = 3), breast (n = 11) and prostate (3)), thyroid cancer (n = 1), cervix cancer (n = 1), sarcomas (osteosarcomas, n = 3), gliomas (n = 2) and liquid tumors (mesotheliomas (n = 7), myelomas (n = 7) and leukemias (n = 3)). As shown in Figures 3A and 3B (in white), sHLA-E was detected in the supernatants of tumor cell lines derived from all origins tested, excepted in the supernatant of ovary tumor, myeloma, leukemias and glioma cell lines (Fig. 3 and data not shown). In our culture conditions (500 000 cells, in 3 ml, during 48 h), the levels of sHLA-E naturally produced by tumor cell lines were ranging from 5 to 400 pg/ml. Highest productions were detected among melanoma, colorectal and kidney tumor cells lines. The frequency analysis of the tumor cell lines able to produce sHLA-E (taking a cut-off value of 5 pg/ml) revealed that about one third of the tumor cell lines produced sHLA-E (24.5%, 24 out of 98) with higher percentages obtained in melanoma (40%, 12 out of 30) and renal cell carcinoma (40%, 4 out of 10) (Fig. 3C).

### Selective regulation of sHLA-E production by cytokines

**Figure 4:**
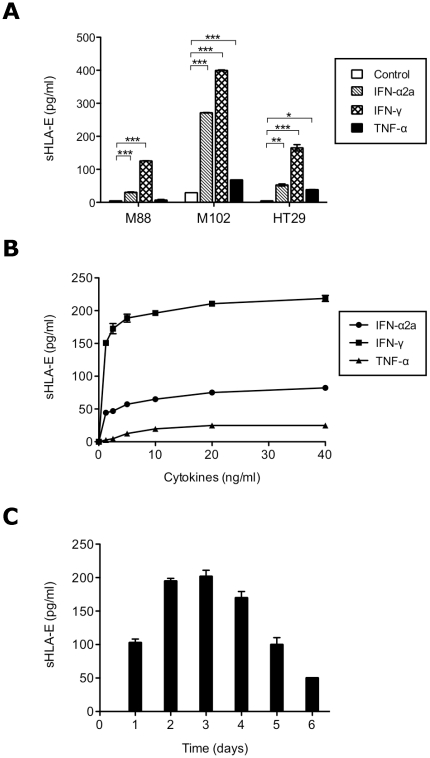
Influence of cytokines on sHLA-E production by tumor cells. A/ sHLA-E detection in supernatants of three tumor cell lines: two melanoma cell lines (M88 and M102) and one colocarcinoma cell line (HT29), treated or not with IFN-α, IFN-γ or TNF-α (10 ng/ml, 48 h). Significant differences between the control and treatment values are indicated (*p<0.05, **p<0.01, ***p<0.001). B/ sHLA-E detection in culture supernatants of M102 treated with serial concentrations of IFN-α, IFN-γ and TNF-α for 48 h. C/ Time course of sHLA-E production in culture supernatant of M102 treated for up to 6 days with 10 ng/ml IFN-γ.

We have previously shown that IFN-γ increased the surface expression of HLA-E and the shedding of soluble HLA-E by melanoma cells. To confirm this result and broaden the panel of tested cytokines, the effect of IL-1β, IL-2, IL-4, IL-6, IL-7, IL-10, IL-12, IL-13, IL-15, IL-23, IFN-α2a, IFN-γ, TNF-α and GM-CSF upon tumor cells lines sHLA-E production was tested. As expected, sHLA-E release was induced or significantly increased in tumor cell supernatants after IFN-γ activation (p<0.0001). The frequency analysis of the tumor cell lines able to produce sHLA-E after IFN-γ treatment doubled (from 24.5% to 49%, 48 out of 98) (Fig. 3C). To a lesser extent, exposure to IFN-α and TNF-α significantly increased the production of sHLA-E by tumor cell lines (p<0.001 and p<0.05 respectively). These results are illustrated in Figure 4A using two melanoma cell lines (M88 and M102) and one colon adenocarcinoma cell line (HT29). Other tested cytokines have no effect on this production. Kinetic analysis and dose-response experiments were performed with IFN-γ, IFN-α and TNF-α. As shown in Figure 4B, sHLA-E production was significantly increased in a dose-dependent manner with the maximal effect observed with 10 ng/ml for the three cytokines. This production was detectable as early as 1 day following cytokines treatment of tumor cells and was maximal after 48 hours (Fig. 4C).

## Discussion

This study provides evidence that serum sHLA-E is significantly increased in patients suffering from melanoma compared to normal individuals, independently of age and gender. We carried this analysis through, using a specific and sensitive ELISA that we developed and validated for the determination of sHLA-E levels in biological fluids. In this large study including 221 individuals, we observed elevated concentrations of serum sHLA-E, as well as higher frequency of sHLA-E positive sera in patients with melanomas compared with healthy donors. Considering the tumor grading, while sHLA-E levels seemed to ascend with advanced disease stages until stage III, we did not find significant association between serum sHLA-E levels and melanoma stage, which may be caused by the inequitable repartition of patients by subgroup. However, if compared to healthy donors, patients with stage III melanoma exhibited highly elevated sHLA-E serum level in terms of both concentration and frequency of positive sera. On the other hand, patients with stage IV disease exhibited lower levels of sHLA-E, which is in accordance with the faint spontaneous expression of HLA-E by metastatic tumor sections that we previously reported by immunohistochemistry [12].

These data emphasized the interest for soluble non-classical HLA-I molecules in malignant diseases. Another non-classical HLA-I molecule, HLA-G, has been identified in diverse malignancies including cancers and has received a lot of attention in recent publications. Clinical studies demonstrated that sHLA-G levels were significantly elevated in sera of patients with malignant melanomas, gliomas, breast and ovarian cancers, non-small-cell-lung cancers (NSCLC), chronic lymphocytic leukemias, B cell and T cell Non-Hodgkin's lymphomas [17]–[21]. Moreover, Zhu et al. demonstrated that serum sHLA-G levels could be a useful indicator in distinguishing colorectal cancers from benign colorectal diseases [22]. Finally, sHLA-G levels were also significantly higher in malignant ascites of ovarian and breast carcinomas than in benign controls [23]. Similarly, elevated levels of the stress-inducible MHC class I chain-related surface glycoproteins, MICA and MICB, have been found to correlate with cancer stages and metastasis in several carcinomas [24]–[26]. Altogether, these studies congruently revealed increased amounts of non classical HLA-I molecules in biological fluids (blood and ascite) of patients suffering from various cancers.

Given the fact that we and others have previously detected sHLA-E in the supernantants of melanoma and colorectal cell lines by Western blot analysis, we investigated, using our designed ELISA, the production of sHLA-E by tumor cell lines derived from major tumor categories, including solid tumors as melanoma, cancers of the breast, colon and kidney and liquid tumors as mesotheliomas and myelomas [12], [27]. We showed that sHLA-E is spontaneously produced by 25% of tested cell lines (24 out of 98), derived from several types of human tumors, especially from melanoma, colorectal and kidney cancers. So, it will be interesting to address the potential predictive and prognostic value of sHLA-E levels in the blood of patients suffering from these cancers.

We have also examined the influence of various cytokines on HLA-E production by tumor cell lines. According to our previous results on melanoma cell lines [12], we showed that IFN-γ induced or increased sHLA-E production, leading to the production of sHLA-E by 50% of the tumor cell lines tested (48 out of 98). Moreover, release of sHLA-E was also driven by IFN-α and TNF-α in a lesser extent. Considering that IFN-γ and IFN-α are high inducers of HLA-I molecules expression, their impact upon sHLA-E production likely reflect the increase of HLA-E expression by tumor cells. Since sHLA-E release by melanoma cells is mainly matrix metalloproteinase-dependant, TNF-α effect on HLA-E production could be due to the ability of TNF-α to promote matrix metalloproteinase expression [12], [28]. These results are consistent with those of Coupel *et al.* showing sHLA-E production by endothelial cells after treatment with IFN-γ and TNF-α [29]. Yet, in opposition to their study, we did not observe any effect of IL-1β treatment on sHLA-E production by tumor cells, probably due to a low expression of IL-1 receptor (IL-1R1) by the tumor cell lines tested in our study. However, as it has been reported that tumor cell lines, including melanoma cell lines, can express IL-1R1, we can postulate that IL-1β, which is known to promote matrix metalloproteinase expression, could increase the production of sHLA-E by IL-1R1 expressing tumor cells [30], [31].

Due to its effect on the proliferation of tumor cells, angiogenesis and its immunomodulatory capacities, IFN-α is used as immunotherapy in the treatment of various solid tumors, as melanoma and renal carcinoma [32]. Therefore, as we show its ability to upregulate sHLA-E production by tumor cell lines, systemic therapy with IFN-α may increase the sHLA-E production in melanoma patients. In this support, IFN-α therapy is associated with elevated sHLA-G serum levels in patients with melanoma [33]. Moreover, it has been reported that γ-irradiation downregulates the surface expression of HLA-G1 on melanoma cells, by enhancing the proteolytic cleavage of this molecule [34]. So, it will be interesting to determine if this mechanism is also observed with HLA-E, which would then be released into the tumor microenvironment and hereby affect the local immunological status.

Independently of the possible mechanism of sHLA-E production, it is important to highlight how the generation of sHLA-E by tumor cells could contribute for immunosurveillance escape. Since interaction of membrane-bound HLA-E with the inhibitory receptors CD94/NKG2-A induced inhibition of NK and T cell responses, the immunosuppressor activity of sHLA-E should be investigated. In support of a potential immunoregulatory fonction, Coupel *et al.* reported that sHLA-E protect endothelial cells from NK-mediated cell lysis [29]. Moreover, sHLA-G and sMICA have been shown to decrease the immune recognition and destruction of tumor cells. sHLA-G, via its interaction with inhibitor receptors ILT-2 and ITL-4, has been shown to inhibit lytic activity of NK cells, to induce apoptosis of CD8^+^ CTL, to affect CD4^+^ alloproliferation and to impair NK/DC crosstalk [35]–[38]. Moreover, the tumor-derived soluble MICA induced endocytosis and degradation of the cognate activatory receptor NKG2-D on tumor-infiltrating lymphocytes, impairing their activation [29], [39]. Altogether, these data emphasized the importance of tumor-derived soluble NKR ligands in providing a tumor microenvironment favoring immune escape. Moreover, it has been reported that sHLA-G are produced *in vitro* as monomeric and multimeric forms and that sHLA-G dimerization augments ILT-2-mediated inhibition of T cell alloresponse [40]. So, the existence of sHLA-E multimers should also be investigated.

In conclusion, the current study provides for the first time evidence of an elevated sHLA-E in sera from melanoma patients, indicating that HLA-E might serve as a clinical marker for the prognosis or prediction of the clinical outcomes of these cancers especially in the context of immunotherapy. Because a sensitive sHLA-E-ELISA has practical advantages for large-scale screening, it could be adopted for routine use in the immunological follow-up of melanomas and other human cancers. Although the function of tumor-derived soluble HLA-E remains to be defined, we can postulate that these molecules could reinforce the host's immune suppression through inhibiting the functions of NK and T cells, and thereby favor the survival of tumor cells. The clinically relevant function of these sHLA-E molecules needs to be carefully analyzed in order to develop appropriate immunotherapeutic strategies.

## Materials and Methods

### Antibodies

MEM-E/07 and MEM-E/08 mAbs (Exbio, Czech Republic), that binds native HLA-E proteins were used for ELISA.

### Peptides and Recombinant soluble HLA

Peptides were purchased from Eurogentec (Angers, France). Purity (>85%) was controlled by reverse-phase high performance liquid chromatography. Diverse HLA and β2-microglobulin recombinant proteins were refolded with the followed indicated synthetic peptides. HLA-E*0101/VMAPRTLVL (HLA-A*0201 signal peptide) and HLA-A*0201/AAGIGILTV (Melan-A_27–35_) monomers were generated by the recombinant protein facility (IFR 26, Nantes). HLA-A*2301/PYLFWLAAI, HLA-B*0702/GILGFVFTL, HLA-B*0801/QAKWRLQTL and HLA-B*2705/HRCQAIRKK monomers were supplied by the tetramer production facility (Ludwig Institute For Cancer Research, Lausanne, Switzerland).

### Patients and specimens

Sera samples were collected from patients with melanoma (n = 127), all with formal consent. Sera from healthy donors (n = 94) were provided by the Etablissement Français du Sang (EFS) (Nantes, France) and used as controls.

### Ethics Statement

Written consents were obtained from all patients and healthy donors. All these studies were approved by the local ethics commmitees “Comité de Protection des Personnes Ouest IV-Nantes” and the “Agence Française de Sécurité Sanitaire des Produits de Santé”.

### Cell lines culture

Melanoma cell lines were established in the GMP Unit of Cellular Therapy and in our laboratory (UMR 892 INSERM/Université de Nantes, France) and belong to the Biocollection PC-U892-NL (CHU Nantes). Colorectal carcinoma cell lines were purchased from ATCC or established in our laboratory UMR 892 INSERM/Université de Nantes (Biocollection PC-U892-FJ, CHU Nantes). Renal carcinoma cell lines were established in INSERM U1016/CNRS UMR 8104, Paris, France) [41]. Breast cancer cell lines were purchased from ATCC or established in our laboratory UMR 892 INSERM/Université de Nantes (Biocollection PC-U892-NG, CHU Nantes). Lung cancer cell lines and mesothelioma cell lines were purchased from ATCC or gifts from M. Grégoire (UMR 892 INSERM/Université de Nantes, France, Biocollection PC-U892-MG, CHU Nantes). Myeloma cells lines were gifts from C. Pellat (UMR 892 INSERM/Université de Nantes, France, Biocollection PC-U892-MA, CHU Nantes). Ovary carcinoma, glioma, leukemia, thyroid, cervix and prostate cancer cell lines were were purchased from ATCC and kindly provided by C. Saï, F. Vallette and F. Paris (UMR 892 INSERM/Université de Nantes, France). Osteosarcoma cells lines were purchased from ATCC and gifts from M. Padrines (EA3822, INSERM U957, Nantes, France). All cell lines were cultured in RPMI or DMEM with 10% of fetal calf serum (FCS, PAA, Austria).

### Tumor supernatants production

For sHLA-E production screening, 500 000 tumor cells were cultured in 6-well plates in 3 ml of 10% FCS-RPMI, supplemented or not with IFN-γ (20 ng/ml). After 48 hours, tumor supernatants were collected, centrifuged 5 min at 2500 g and kept frozen before testing in ELISA.

The impact of others cytokines under sHLA-E production, has initially been tested at 20 ng/ml during 48 hours with two melanomas cell lines (M88 and M102) and one colorectal adenocarcinoma cell line (HT29). Dose-response and kinetics assessments of IFN-γ, IFN-α2a and TNF-α were secondarily performed as described (concentration ranging from 40 to 0,02 ng/ml during 1 to 6 days) with these three tumors cell lines.

### Detection of sHLA-E by ELISA

Nunc-Immuno MaxiSorp Microtiter plates were coated (50 µL/well) with MEM-E/08 mAb at 1 µg/ml in carbonate/bicarbonate buffer (CO_3_HNa 35 mM, CO_3_Na_2_ 15 mM, pH 9.5) overnight at 4°C. After four washes with PBS-0.05% Tween 20 (200 µL/well), plates were saturated with PBS containing 10% FCS for 2 h at room temperature (200 µL/well). After four washes, the biological samples (50 µl/well optionally diluted in saturation buffer) were added (in triplicate) and incubated for 2 h at room temperature. Culture supernatants were assayed undiluted while six doubling dilutions of sera were used. The detecting biotinylated MEM-E/07 mAb diluted at 1 µg/ml in saturation buffer was added after four washes (50 µL/well) and incubated again for 2 h at room temperature. Plates were washed four times and incubated with strepta-HRP reagent (BD pharmingen) diluted at 1/1000 in saturation buffer for 1 h at room temperature. Finally, plates were washed four times and incubated with substrate (3,3′,5,5′-tetramethylbenzidine liquid, Sigma, ST Qentin Fallavier, France) for 30 min at room temperature in the dark (100 µL/well). The reaction was stopped by addition of 100 µL/well of 1 M H_3_PO_4_. Absorbance was measured at 450 nm with a Thermo Scientific Multiskan EX. Analysis of the standard curve and interpolation of samples concentrations were performed using Prism 5 Software (GraphPad Software Inc., La Jolla, CA, USA).

### Statistical analysis

Sera of cancer patients were compared with sera of healthy donors. According to non-parametric distribution of sHLA-E serum levels, data were presented as means, medians, ranges and percentages of positive sHLA-E sera. For the general comparison of two groups, statistical analysis was performed by Mann-Whitney U test. Distribution of concentrations across stage was assessed using Kruskal-Wallis test. To compare the frequency of positive sHLA-E sera, the Fisher test was used. Statistical analysis of the modulatory effect of cytokines on sHLA-E production by tumor cell lines was performed by one-way ANOVA and followed by post hoc Bonferroni test. A P-value<0.05 was considered statistically significant. All statistical analyses were performed using Prism 5 Software (GraphPad Software Inc., La Jolla, CA, USA).
